# Nutrition Risk Screening and Related Factors Analysis of Non-hospitalized Cancer Survivors: A Nationwide Online Survey in China

**DOI:** 10.3389/fnut.2022.920714

**Published:** 2022-06-21

**Authors:** Fang Wang, Qi Dong, Kang Yu, Rong-rong Li, Ji Fu, Jia-yu Guo, Chun-wei Li

**Affiliations:** Department of Clinical Nutrition, Peking Union Medical College Hospital, Chinese Academy of Medical Sciences (CAMS) and Peking Union Medical College (PUMC), Beijing, China

**Keywords:** non-hospitalized cancer survivors, online survey, oncology, nutritional risk, nutrition intervention

## Abstract

**Purposes:**

This study investigated the nutritional problems and risks of Chinese non-hospitalized cancer survivors through an online survey.

**Methods:**

The survey included nutritional and clinical questions distributed to non-hospitalized cancer survivors. All data were screened and analyzed with strict quality control. Nutrition Risk Screening-2002 (NRS-2002) was adopted and the related factors were analyzed.

**Results:**

Six thousand six hundred eighty-five questionnaires were included. The prevalence of nutritional risk was 33.9%, which varied according to age, sex, cancer type, TNM staging, oncologic treatment, time interval since last treatment, etc. In the regression analysis, nutritional risk was associated with age, TNM staging, and nutrition support. Patients with leukemia and digestive cancer had the highest NRS-2002 score (3.33 ± 1.45 and 3.25 ± 1.61); the prevalence of nutritional risk (NRS-2002 ≥ 3) was 66.7 and 55.1%, respectively. Patients with a higher TNM stage had higher NRS-2002 scores in non-digestive cancer, which was not seen in digestive cancer. Among digestive, bone, nervous, and respiratory cancer patients, the NRS-2002 score mainly consisted of “impaired nutritional status,” which coincided with the “disease severity score” in leukemia patients. Nutrition intervention was achieved in 79.7 and 15.2% of patients with nutritional risk and no risk. Of the patients, 60.3% exhibited confusion about nutritional problems, but only 25.1% had professional counseling.

**Conclusions:**

Regular nutritional risk screening, assessment, and monitoring are needed to cover non-hospitalized cancer survivors to provide nutrition intervention for better clinical outcome and quality of life. By online survey, the nutritional risk of non-hospitalized cancer survivors was found high in China, but the nutrition support or professional consultation were not desirable. The composition of nutritional risk should also be aware of.

## Introduction

It is estimated that about 10 million deaths from cancer in 2020 worldwide (1). Malnutrition is commonly seen among cancer patients and account for 10–20% of cancer death (2). Weight loss and dietary reduction promote the occurrence of malnutrition in cancer patients, thus affecting oncologic treatments, overall survival time, and quality of life (3–5). Standardized nutritional support starts with nutritional risk screening, which is an essential first step in structured process of nutrition care, aims to identify nutritional risks, and to provide the appropriate amount of nutritional support for improving patient outcomes (6–8). European guidelines recommend immediate nutritional screening for all patients after cancer detection (9, 10). The Investigation on Nutrition Status and Its Clinical Outcome of Common Cancer (INSCOC), which was conducted in 22 cities covering more than 80 hospitals in China, found that 26.1, 32.1, and 22.2% of in-patients had severe, moderate, and mild malnutrition (11). However, it is worth noting that a large proportion (~75%) of cancer survivors are non-hospitalized, who are either in remission or between treatment cycles. To date, large sample surveys investigating the nutritional status of this population as well as their nutrition-related clinical problems remain scarce. The nutritional risk and demand of these non-hospitalized cancer survivors should be investigated, therefore there is an urgent requirement for action to fill this blank. Since it is hard for doctors or dietitians to reach this population and provide nutrition service, with the support of online survey, we can assess the nutritional status on non-hospitalized patients in a wide range.

Malnutrition has adverse effect on quality of life and overall survival (5, 12), thus understanding the nutritional status and identification of the nutritional risk would bring benefit to provide appropriate nutrition support in time, so as to improve the clinical outcome. Among the numerous nutrition screening tools used in clinical practice, the nutritional risk screening 2002 (NRS-2002) is a tool developed by Kondrup (8) and an ESPEN working group in 2002, which is based on the outcome observed in randomized controlled trials and identifies patients who are likely to benefit from nutritional support by an improved clinical outcome. As recommended by ESPEN, NRS-2002 is content valid and could be done by various health providers. Even though NRS-2002 is not designed for cancer patients, the high validity is also confirmed by cancer patients, that participants identified with nutritional risk by NRS-2002 developed a decreased overall survival and worse clinical outcome (13, 14). Moreover, the secondary analysis of EFFORT study showed that for cancer patients at high risk, personalized nutrition intervention resulted in increased survival and better quality of life (15). The study suggests that NRS-2002 is also suitable for out-patients and community-living cancer patients (16). Moreover, key influencing factors for nutritional risk includes clinical diagnosis, oncologic treatment, gender, gastrointestinal symptoms, education, and income, all of which can also be collected by online survey.

During the era of COVID-19, in which face-to-face interviews are limited, this study investigated the nutritional status targeting non-hospitalized cancer survivors who are in remission or between treatment cycles by an online questionnaire, and hypothesized the nutritional status of this population was of concern and required attention by physicians and dietitians. Moreover, risk factors and composition of nutritional risk will be analyzed.

## Materials and Methods

### Subjects

These questionnaires were distributed through a network push by three large social management institutions for cancer patients, which have tens of thousands of registered cancer patients across the country. This ensured that large-scale group surveys could be promoted in a short span of time. The administrators of the organizations emailed or sent WeChat messages containing a study invitation to registered patients; “if you are a cancer patient who is currently in the treatment interval or at the end of treatment, you may consider completing the following questionnaire survey.” If the patients opted to participate, they will provide an electronic signature on the consent. Then a link to an online questionnaire will be sent to the patients. The time frame for recruitment was between February 2020 and June 2020. The inclusion criteria were as follows: (1) age ≥ 18y, regardless of sex; (2) patients with pathological or clinical diagnosis of cancer (unlimited tumor types); (3) non-hospitalized survivors who were in remission or in between treatment cycles; (4) patients who voluntarily participated in the survey. The exclusion criteria were as follows was: (1) patients who refused to participate.

The protocol was approved by the Human Ethics Committee of Peking Union Medical College Hospital (No. ZS-2601); all participants provided written informed consent. The study was registered at ClinicalTrials.gov (NCT 04778540).

### Questionnaire Design and Data Collection

The survey aimed to identify the prevalence of nutritional risks in non-hospitalized cancer survivors and describe their nutritional status and support requirements. The questionnaire was developed according to criteria of NRS-2002, including age, nutritional status (percentage of weight loss, general condition, and recent food intake), and disease severity (diagnosis and stage) (8). Moreover, based on literature review, the general risk factors, such as education, residence, payment methods, recent treatment, the time interval between the survey taken and last oncological treatment, and current nutrition support were also listed in the questionnaire. The design followed the principles of voluntariness, acceptability, objectivity, and non-orientation. Patients were first introduced to the study purpose and confidentiality principles. Objective and closed questions were listed at the beginning, followed by subjective questions, such as appetite, food intake, gastrointestinal symptoms, and access to nutritional support. Factual personal questions, such as education and insurance, were placed at the end. The survey was concise and could be answered within 10-min timeframe. Next, the multi-disciplinary research team of the Nationwide Online Survey on Nutritional Risk and Clinical Outcome of Non-hospitalized Cancer Patients (NOS-NOC), consisting of clinical dietitians, oncologists, epidemiologists, psychologists, and nurses, discussed the questions summarized and evidence from literature reviews with a focus on implementation and domains for nutritional risk screening. During this process, the questions were modified to be more understandable of patients and added with attitudes and perspectives on nutrition, such as the tendency and frequency of nutrition department visits.

The NRS-2002 score is calculated by adding the nutritional status impaired score (0–3) to the severity of disease score (0–3) plus a score of 1 for patients' age ≥70 years. The total NRS-2002 score ranges from 0 to 7. The nutritional status impaired score is determined by quartiles of decreased oral food intake in the previous week, the presence of weight loss of at least 5% during the previous 1–3 months, and a low body mass index (BMI) combined the impaired general condition (7). In this study, we adapted Chinese BMI criteria (normal range 18.5 ≤ BMI < 24.0) established by Chinese Obese Working group, which is according to population research (17). Weight loss and reduction of food intake were self-reported. The severity of disease was evaluated based on the patient's choice of whether there is severe condition listed in NRS-2002 criteria, and categorized as none, slight, moderate, or severe, and converted to scores of 0-3. According to the recommendations by ESPEN Screening Guideline, an NRS score ≥ 3 means nutritionally at risk and a NRS score <3 means no at nutritional risk (7).

**Figure 1:**
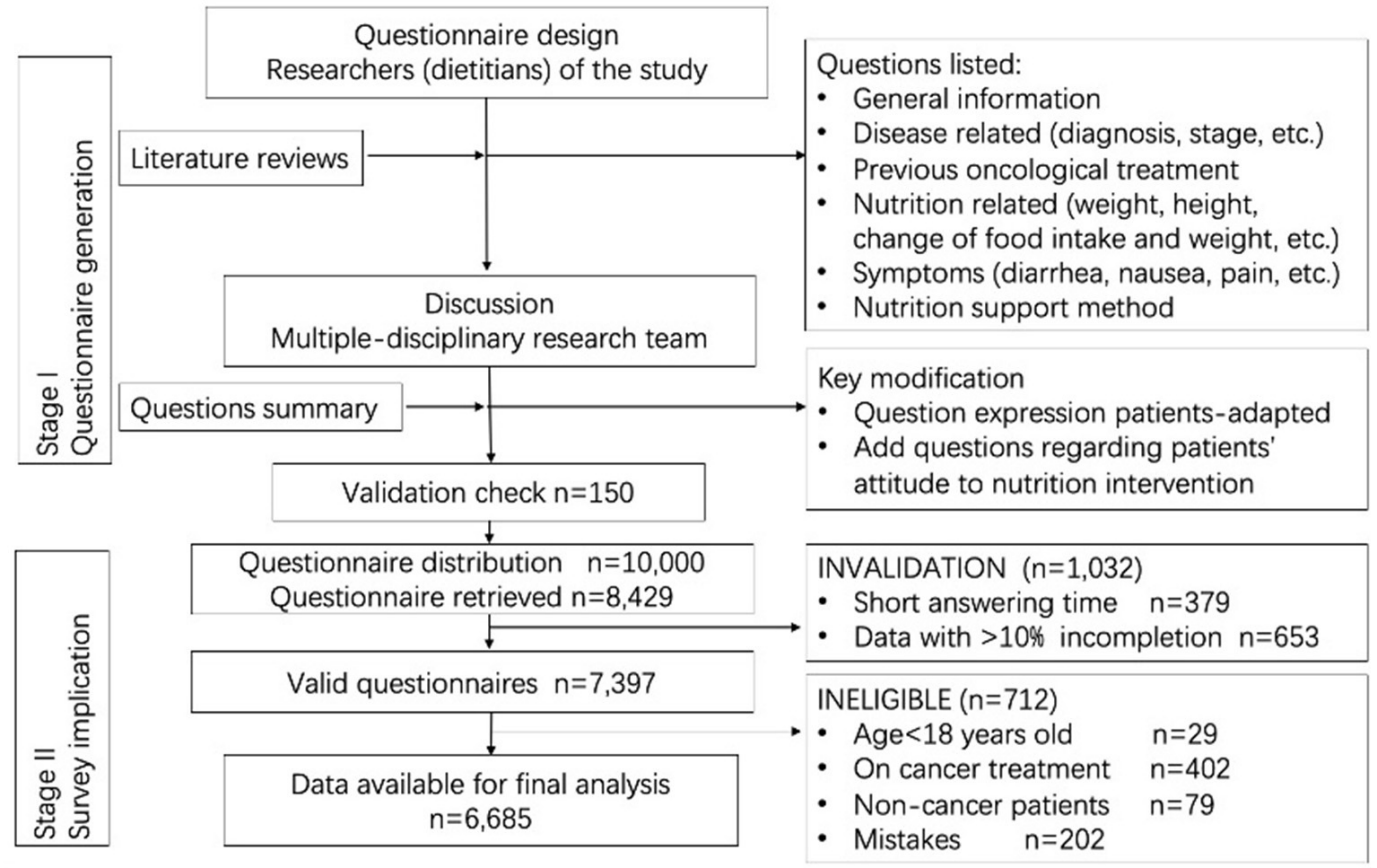
Flowsheet.

Investigators distributed 150 questionnaires to perform a pretest in order to assess reliability and validity and retrieved 121 (response rate 80.7%). Researchers conducted telephone follow-ups to evaluate the participant's nutritional status, compared it with their answers to assess the agreement. Those with a completion rate of more than 90% and agreement of more than 95% were considered validated and reliable. Finally 114 questionnaires were thought to be qualified (94.2%). Moreover, the researchers also asked participants whether they felt the questions were too long or complicated and found good acceptability. Hence, the questionnaire was regarded as reasonable, valid, and reliable (see Figure 1 Flowsheet).

### Quality Control

Our research team recruited eight registered dietitians (RDs) from the aforementioned social management institutions for cancer patients; they underwent a 1-week research training for conducting the purpose, methods, procedure, and quality control requirements of this investigation. Furthermore, they became the investigative assistants of the research team for data review and logical inspection.

First, the questionnaires were scanned and filtered according to the inclusion and exclusion criteria. Next, troubleshooting was conducted, which was divided into an automatic program error check and a manual check. Network engineers set up initial graphic verification to intercept the questions answered by a machine. Then, SMS verification was carried out to ensure the real existence of the phone number. At the same time, each IP address was limited to only one submission. The average answering time of this questionnaire was about 300 seconds, depending on the pretest; only those with more than 180 s of answering time were considered valid. Common sense errors, such as selecting EN and PN at the same time, choosing four kinds of cancer at the same time, obviously wrong height and weight reports, were excluded. Additionally, the RDs participated in the audit with 30 working days and a total of more than 800 h. As performed, 20% of randomly selected questionnaires were checked by phone to confirm the accuracy.

### Statistical Analysis

Categorical data were described as relative frequencies; quantitative variables were described as means and standard deviations. According to age, gender, different sites of the primary tumor, and staging, we divided the data into different subgroups; only subgroups with more than 30 participants were included for statistical validity. The differences in nutritional risk scores were compared among the subgroups using *t*-test or analysis of variance (ANOVA). The NRS-2002 score was treated as either a continuous or categorical variable for a nutritional risk classification threshold of 3 (NRS-2002 < 3, NRS-2002 ≥ 3); this was to classify the subjects with or without nutritional risk (7). To investigate the reason for nutritional risk, we further analyzed the composition of the NRS-2002 score. The association between NRS-2002 score and age, gender, site of tumor, cancer stage, therapy, interval of oncological treatment, and symptoms were investigated by univariable and multivariable linear regression analysis. NRS-2002 was alternatively treated as a continuous variable, or as categorical toward a classification threshold of 3 (NRS <3, NRS ≥3). The data were entered into SPSS version 24.0; two-sided *P* < 0.05 was considered significant.

## Results

### Demographic Characteristics

A total of 10,000 questionnaires were sent during the recruitment period and received respondents of 8,429 (84.3%). 379 and 653 questionnaires were excluded because of the short answering time and incompletion, respectively. According to the inclusion and exclusion criteria, a total of 712 patients were excluded as follows: 29 patients aged < 18 years; 402 patients currently undergoing oncological treatment; 79 non-cancer patients; and 202 questionnaires found with common sense or logical mistakes. Finally, 6,685 questionnaires were entered final analysis (see Figure 1 for the flowsheet).

The online survey enrolled participants from 31 provinces, autonomous regions, and municipalities of China. The demographic characteristics are listed in Supplementary Table 1; more females than males as well as more urban residences than rural areas were recorded. The most frequently reported case was lung cancer (2,492, 37.3%), while others included esophageal (246, 3.7%), gastric (402, 6.0%), liver (158, 2.4%), breast (1,051, 15.7%), ovarian (706, 10.6%), and colorectal cancer (277, 4.2%); 1,219 cases were categorized as digestive system cancer, while 5,466 cases were of non-digestive system cancer. The majority of participants suffered from diarrhea (1,416, 21.2%), abdominal distension (1,501, 22.5%), nausea or vomiting (2,864, 42.8%), acid reflux or heartburn (1,062, 15.9%), and constipation (2,230, 33.3%) for at least 1 week.

### Weight Change

The following were revealed: 610 (9.1%) participants being underweight (BMI < 18.5 kg/m^2^); 1,693 (25.3%) being overweight; and 575 (8.6%) being obese. A total of 1,089 (16.3%), 417 (6.2%), and 327 (4.9%) patients had > 5% weight loss during the past 3, 2, and 1 months, respectively, while 279 (4.2%) patients reported visible wasting. More than 10% of patients with laryngeal, esophageal, gastric, and bone cancer had > 5% weight loss within 1 month (Table 1).

**Table 1:** BMI, weight, and dietary reduction, NRS-2002 score of the participants.

**Cancer type**	**N**	**NRS-2002 score**			**BMI**				**Weight change**				**Dietary reduction**		
		**Average score**	**<3** **N (%)**	**≥3** **N (%)**	**<18.5** **N (%)**	**18.5–23.9** **N (%)**	**24–27.9** **N (%)**	**≥28** **N (%)**	**>5% weight loss within 3 months** **N (%)**	**>5% weight loss within 2 months** **N (%)**	**>5% weight loss within 1 month** **N (%)**	**Visibly wasting away** **N (%)**	**25–50% dietary reduction in the recent 1 week** **N (%)**	**51–75% dietary reduction in the recent 1 week** **N (%)**	**76–100% dietary reduction in the recent 1 week** **N (%)**
Lung	2454	1.89 ± 1.32	1709 (69.6)	745 (30.4)	229 (9.3)	1367 (55.7)	640 (26.1)	218 (8.9)	348 (14.2)	134 (5.5%)	99 (4.0)	114 (4.6)	274 (11.2)	209 (8.5)	125 (5.1)
Other respiratory system	50	1.74 ± 1.1	34 (68.0)	16 (32.0)	1 (2.0)	31 (62.0)	12 (24.0)	6 (12.0)	11 (22.0)	7 (14.0)	3 (6.0)	3 (6.0)	10 (20.0)	13 (26.0)	4 (8.0)
Oral	25	2.56 ± 1.29	10 (40.0)	15 (60.0)	1 (4.0)	16 (64.0)	7 (28.0)	1 (4.0)	6 (24.0)	5 (20.0)	1 (4.0)	2 (8.0)	4 (16.0)	5 (20.0)	2 (8.0)
Gastric	402	2.78 ± 1.38	166 (41.3)	236 (58.7)	56 (13.9)	254 (63.2)	70 (17.4)	22 (5.5)	98 (24.4)	47 (11.7)	40 (10.0)	21 (5.2)	58 (14.4)	65 (16.2)	47 (11.7)
Esophageal	245	2.62 ± 1.3	96 (39.2)	149 (60.8)	30 (12.2)	152 (62.0)	43 (17.6)	20 (8.2)	68 (27.8)	24 (10.0)	29 (11.8)	12 (4.9)	48 (19.6)	41 (16.7)	16 (6.5)
Liver	155	2.53 ± 1.21	73 (47.1)	82 (52.9)	16 (10.3)	96 (61.9)	33 (21.3)	10 (6.)	39 (25.2)	14 (9.0)	15 (10.0)	11 (7.1)	25 (16.1)	24 (15.5)	12 (7.7)
Colorectal	271	2.58 ± 1.26	138 (50.9)	133 (49.1)	29 (10.7)	156 (57.6)	67 (24.7)	19 (7.0)	56 (20.7)	18 (6.6)	20 (7.4)	15 (5.5)	38 (14.0)	34 (12.5)	16 (5.9)
Pancreas	45	2.59 ± 1.31	21 (46.7)	24 (53.3)	8 (17.8)	27 (60.0)	7 (15.6)	3 (6.7)	11 (24.4)	4 (8.9)	3 (6.7)	4 (8.9)	5 (11.1)	7 (15.6)	5 (11.1)
Gallbladder	27	2.56 ± 1.25	15 (55.6)	12 (44.4)	4 (14.8)	13 (48.1)	9 (33.3)	1 (3.7)	6 (22.2)	3 (11.1)	0	2 (7.4)	2 (7.4)	4 (14.8)	0
Bile duct	25	2.56 ± 1.29	11 (44.0)	14 (56.0)	3 (12.0)	17 (68.0)	4 (16.0)	1 (4.0)	8 (32.0)	2 (8.0)	1 (4.0)	2 (8.0)	3 (12.0)	3 (12.0)	1 (4.0)
Other digestive system	24	2.38 ± 1.62	17 (71.0)	7 (29.0)	5 (17.0)	8 (33.0)	8 (33.0)	3 (13.0)	5 (21.0)	0	1 (4.0)	0	3 (12.5)	0	2 (8.3)
Kidney	78	2.36 ± 1.1	51 (65.4)	27 (34.6)	3 (3.8)	38 (48.7)	18 (23.1)	19 (24.4)	11 (14.1)	3 (3.8)	3 (3.8)	1 (1.2)	5 (6.4)	6 (7.7)	4 (5.1)
Ureter	60	1.87 ± 1.17	39 (65.0)	21 (35.0)	4 (6.7)	40 (66.7)	12 (20.0)	4 (6.7)	12 (20.0)	9 (15.0)	4 (6.7)	3 (5.0)	5 (8.3)	4 (6.7)	1 (1.7)
Bladder	64	2.42 ± 1.31	36 (56.3)	28 (43.8)	12 (18.)	31 (48.4)	14 (21.9)	7 (10.9)	12 (18.8)	8 (12.5)	4 (6.3)	2 (3.1)	5 (7.8)	4 (6.3)	2 (3.1)
Prostate	67	2.13 ± 1.03	40 (59.7)	27 (40.3)	6 (9.0)	46 (68.7)	12 (17.9)	3 (4.5)	11 (16.4)	5 (7.5)	6 (9.0)	2 (3.0)	12 (17.9)	2 (3.0)	2 (3.0)
Other urinary system	11	2.0 ± 1.34	8 (72.7)	3 (27.3)	0	5 (45.4)	4 (36.4)	2 (18.2)	4 (36.4)	0	0	1 (9.1)	1 (9.1)	1 (9.1)	1 (9.1)
Leukemia	33	3.33 ± 1.45	11 (33.3)	22 (67.7)	9 (27.3)	11 (33.3)	11 (33.3)	2 (6.1)	3 (9.1)	9 (27.3)	3 (9.1)	0	1 (3.0)	3 (9.1)	3 (9.1)
Lymphoma	53	2.68 ± 1.14	26 (49.1)	27 (50.9)	6 (11.3)	21 (39.6)	20 (37.8)	6 (11.3)	14 (26.4)	5 (9.4)	2 (3.8)	2 (3.8)	9 (17.0)	3 ( 5.7%)	2 (3.8)
Other blood system	20	2.55 ± 1.1	11 (55.0)	9 (45.0)	1 (5.0)	7 (35.0)	6 (3.0%)	6 (3.0)	4 (20.0)	2 (10.0)	1 (5.0)	3 (15.0)	1 (5.0)	1 (5.0)	1 (5.0)
Bone	120	2.34 ± 1.49	64 (53.3)	56 (46.7)	18 (15.0)	58 (48.3)	34 (28.3)	10 (8.3)	25 (20.8)	10 (8.3)	14 (11.7)	11(9.2)	21 (7.5)	16 (13.3)	10 (8.3)
Skin	150	1.82 ± 1.18	103 (68.7)	47(31.3)	16 (10.7)	81 (54.0)	38 (25.3)	15 (10.0)	29 (19.3)	14 (9.3)	7 (4.7)	5 (3.3)	19 (12.7)	9 (6.0)	7 (4.7)
Cerebral	79	2.2 ± 1.52	44 (55.7)	35 (44.3)	10 (12.7)	46 (58.2)	12 (15.2)	11 (16.5)	21 (26.6)	11 (13.9)	7 (8.9)	5 (6.3)	7 (8.9)	12 (15.2)	4 (5.1)
Uterus	246	2.45 ± 1.24	135 (54.9)	111 (45.1)	27 (11.0)	146 (59.3)	58 (23.6)	15 (6.1)	64 (26.0)	16 (6.5)	17 (6.9)	9 (3.7)	31 (12.6)	27 (11.0)	14 (5.7)
Ovary	698	2.29 ± 1.05	491 (70.3)	207 (29.7)	42 (6.0)	403 (57.7)	193 (27.7)	60 (8.6)	88 (12.6)	26 (3.7)	14 (2.0)	22 (3.2)	74 (10.6)	42 (6.0)	26 (3.7)
Other gynecologic	124	2.12 ± 1.17	89 (71.8)	35 (28.2)	13 (10.5)	71 (57.3)	34 (27.4)	6 (4.8)	15 (12.1)	6 (4.8)	2 (1.6)	3 (2.4)	8 (6.5)	6 (4.8)	5 (4.0)
Breast	1,033	1.42 ± 0.94	885 (85.7)	148 (14.3)	56 (5.4)	586 (56.7)	297 (28.8)	94 (9.1)	104 (10.1)	27 (2.6)	24 (2.3)	19 (1.8)	61 (5.9)	41 (4.0)	21 (2.0)
Nasopharynx	112	1.66 ± 1.14	86 (76.8)	26 (23.2)	5 (4.5)	70 (62.5)	26 (23.2)	11 (9.8)	15 (13.4)	7 (6.3)	4 (3.6)	4 (3.6)	10 (8.9)	13 (11.6)	4 (3.6)
Larynx	14	2.07 ± 0.96	8 (57.1)	6 (42.9)	0	10 (71.4)	4 (28.6)	0	1 (7.1)	1 (7.1)	3 (21.4)	1 (7.1)	1 (7.1)	3 (21.4)	0
F		24.19													
*P*		<0.01													

### Dietary Intake and Nutrition Support

This part was based on the following parameters: recent dietary change and any nutritional support received recently, such as oral nutritional supplements, tube feeding, or parenteral nutrition. As a result, 337 (5.0%) patients reported more than 75% dietary reduction, while 598 (8.9%) and 741 (11.1%) had a reduction of 50–75 and 25–50%, respectively. In addition, due to the direct impact on food intake, digestion, and absorption, digestive system cancer contributed to 38.6% of all food intake reduction cases (Table 1).

In terms of nutritional support, 79.7% of patients with nutritional risk and 15.2% of patients with no risk received nutritional support. A total of 5,953 patients (89.1%) tolerated oral meals. Among them, oral nutritional supplements (ONS) were used in 1,249 cases; nasal enteral nutrition was needed in 365 cases; and parenteral nutrition was needed in 385 cases. Lastly, 672 and 60 cases depended on total nasal feeding and total parenteral nutrition, respectively.

### Severity of Disease

This part was based on the questions regarding whether there was any severe pneumonia, dialysis, bone marrow transplantation, stroke, head injury, or organ dysfunction, and evaluated by the experienced oncologist and clinical dietitians in the research team. Patients with hematologic malignancies, severe pneumonia, or stroke were thought to have a score of 2; patients reported to have bone marrow transplantation or head injury were regarded as having a score of 3. If there is nothing severe complications, the score would be rated as 1 score due to cancer. As a result, 19 (0.1%), 72 (1.1%), and 6,604 (98.8%) patients had score of 3, 2, and 1.

### Nutrition Risk Screening

Nutritional risk screening was based on the following parameters: age, recent changes in weight, food intake, primary tumor site, diagnosis, and disease severity. A total of 2,268 patients (33.9%) had an NRS-2002 score ≥ 3; the prevalence of nutritional risk varied among cancer types. Leukemia patients ranked the highest by both the average score (3.33 ± 1.45) and the percentage of nutritional risk (66.7%), followed by digestive system cancers with a high incidence of nutritional risk (55.1%) and a high NRS-2002 score. Leukemia and digestive system cancer were the only two diseases with more than half of the patients at nutritional risk. Patients with breast cancer had the lowest NRS-2002 score and percentage (14.3%) with NRS ≥ 3 (see Table 1, Supplementary Figure 1).

#### Age and Sex

The NRS-2002 scores varied significantly among the different age groups. For <45, 45–65, 65–85, and > 85-year-old groups, the NRS-2002 scores were 1.72 ± 1.12, 1.83 ± 1.43, 2.39 ± 1.37, and 2.75 ± 1.72, respectively (*P* < 0.05). Subgroup analysis showed that nutritional risk increased with age in patients with lung and colorectal cancers. For gastric, esophageal, skin, and breast cancer, the NRS-2002 score was like shaped like a “U,” that is, high at < 45 years old, decreased at 45–65 years old, and then increased at > 65 years old. Gastric and lung cancer patients in the > 85 years old group had the highest NRS-2002 score (4.02 ± 1.01 and 4.10 ± 1.41, respectively), while breast cancer patients aged 45–65 years old had the lowest score (1.46 ± 0.94). The NRS-2002 scores of males were significantly higher than females, which were 1.96 ± 2.09 and 1.25 ± 1.30, respectively (*P* = 0.001) (Table 2).

**Table 2:** NRS-2002 score of subjects according to different age and TNM staging.

**Cancer type**	**Age**						**TNM staging**					
	** <45**	**45–65**	**65–85**	**>85**	**F**	***P***	**I**	**II**	**III**	**IV**	**F**	***P***
Lung	1.8 ± 1.25	1.88 ± 1.27	2.69 ± 1.51	4 ± 1.0	84.29	<0.01	1.57 ± 1.01	2.05 ± 1.32	1.88 ± 1.29	2.16 ± 1.41	10.58	<0.01
Gastric	3.13 ± 1.33	2.96 ± 1.31	3.73 ± 1.51	4 ± 1.41	6.47	0.002	2.73 ± 1.28	3.15 ± 1.36	3.31 ± 1.29	3.19 ± 1.49	2.19	0.069
Esophageal	3.1 ± 1.28	2.96 ± 1.35	3.02 ± 1.39	0	0.19	0.823	2.48 ± 1.24	3.18 ± 1.24	3.31 ± 1.39	2.52 ± 1.33	2.28	0.105
Liver	–	–	–	–	–	–	2.53 ± 1.12	2.93 ± 1.24	2.95 ± 1.15	3.25 ± 1.29	0	0.945
Colorectal	2.37 ± 1.11	2.79 ± 1.25	3.35 ± 1.49	0	7.63	0.001	2.55 ± 1.24	2.76 ± 1.04	2.89 ± 1.46	3.14 ± 1.59	1.68	0.172
Bone	–											
Skin	2.19 ± 1.38	1.68 ± 1.05	4 ± 0	0	4.39	0.039	1.53 ± 1.03	2.31 ± 1.32	1.71 ± 1.11	1 ± 0	8.71	0.004
Uterus	2.7 ± 1.36	2.72 ± 1.24	3.25 ± 1.82	0	0.01	0.904	2.32 ± 1.21	3.01 ± 1.31	3 ± 1.3	3.19 ± 1.05	7.81	0.001
Ovary	2.35 ± 1.12	2.4 ± 1.11	2.69 ± 0.87	0	1.29	0.275	2.2 ± 0.88	2.62 ± 1.22	2.37 ± 1.06	2.66 ± 1.27	4.63	0.003
Other gynecologic	2.33 ± 1.28	2.2 ± 1.2	2.11 ± 0.78	0	0.32	0.574	–	–	–	–	–	–
Breast	1.54 ± 1.01	1.46 ± 0.94	2.43 ± 1.43	0	23.06	<0.01	1.4 ± 0.83	1.48 ± 0.96	1.58 ± 1.02	1.74 ± 1.22	4.15	0.002
Nasopharynx	1.61 ± 1.05	1.74 ± 1.14	3.2 ± 1.55	0	0.34	0.559	1.72 ± 1.02	2.08 ± 1.27	1.87 ± 1.41	1.31 ± 0.63	0.42	0.519

#### TNM Staging and Oncological Treatment

The NRS-2002 scores for stages I to IV were 1.80 ± 1.06, 2.03 ± 1.25, 2.03 ± 1.24, and 2.08 ± 1.40, respectively (*P* = 0.001), which increased along with the disease stage. A positive relationship between the NRS-2002 score and the stage of lung, skin, uterine, ovarian, and breast cancer was observed. However, the NRS-2002 scores for digestive system and nasopharyngeal carcinoma, which were closely associated with food intake, did not show significant differences between the different stages (Table 2). In addition, there was a significant difference in nutritional risk among the different oncological treatments and the time interval since last oncological treatment. The NRS-2002 score was higher in patients who had bone marrow transplantation (4.32 ± 1.27) than in those had surgery plus radio/chemotherapy, surgery, and radio/chemotherapy, with scores of 2.14 ± 1.27, 2.10 ± 1.20, and 1.94 ± 1.31, respectively (*P* = 0.002). Moreover, the combined treatment was more likely to be at nutritional risk than monotherapy. According to the time interval since last oncological treatment, we divided the participants into <1, 1–6, 6–12, and >12 months, and found the NRS-2002 score were 3.04 ± 1.32, 1.50 ± 1.04, 1.44 ± 0.92, and 1.01 ± 0.71, respectively (*P* = 0.032).

#### Medical Insurance and Education

Overall, the patients with commercial insurance had the lowest NRS-2002 score (2.25 ± 1.22), followed by self-paid and urban resident insurance (2.48 ± 1.45 and 2.66 ± 1.31, respectively); those with rural cooperative medical insurance had the highest score (2.86 ± 1.34, *P* = 0.002). In terms of education level, the NRS-2002 score of patients with a bachelor's degree or above was 1.91 ± 1.22, whereas those with a high school diploma was 2.32 ± 1.28; on the other hand, those with a primary school diploma and below was the highest (2.65 ± 1.45, *P* < 0.001).

#### Composition of NRS-2002 Score

**Figure 2:**
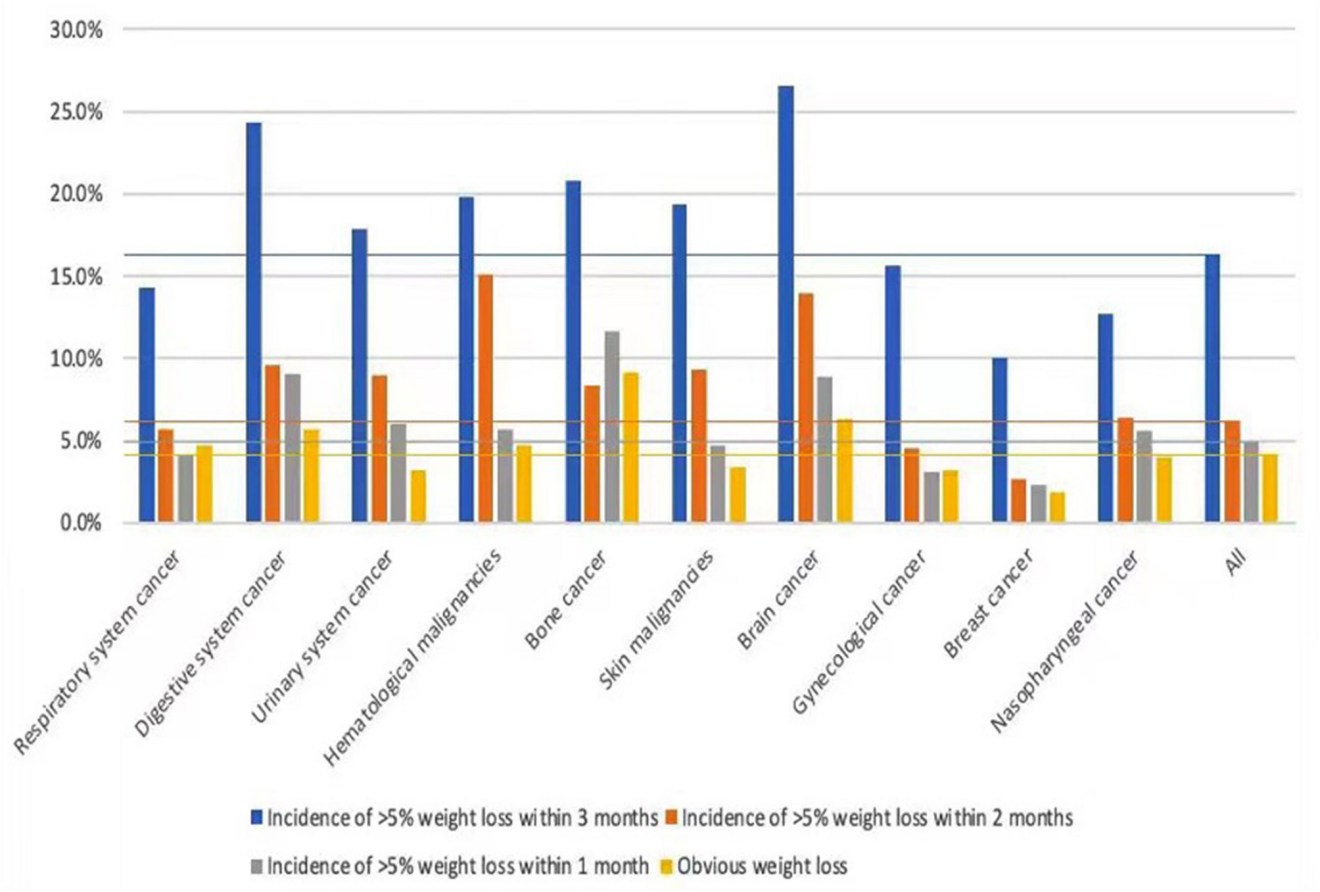
Incidence of different degree of weight loss.

**Figure 3:**
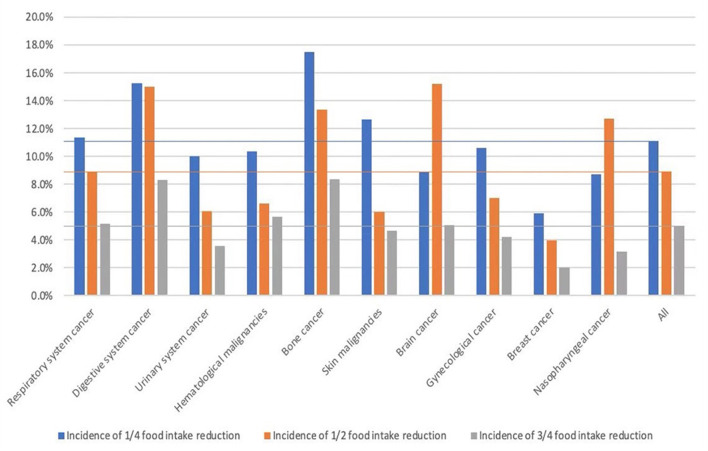
Incidence of different degree of food intake reduction.

We further analyzed the composition of the NRS-2002 score as a source of nutritional risk. Subgroup analysis showed that groups with nutritional impairment, which accounted for ≥ 50% of the total NRS-2002 score, were digestive system (71.4%), bone (67.6%), nervous system (64.2%), and respiratory system cancers (51.2%). In those with hematological cancer, only 34.4% of patients had a proportion of nutritional impairment exceeding 50% of the total score, thereby indicating that the nutritional risk of these patients was mainly contributed by disease severity. Figures 2, 3 shows the percentage of different degrees of weight loss and different degrees of dietary reduction. Compared to the average level, the percentage of severe weight loss (incidence of > 5% weight loss within 1 month and obvious weight loss) in respiratory system, digestive system, hematological, bone, brain, and nasopharyngeal cancers was significantly higher. Moreover, the percentage of more than 50% food reduction in digestive system, bone, brain, and nasopharyngeal cancers exceeded the average level. Moreover, in order to further evaluate the relationship between nutritional impairment and cancer advancement, we conducted the correlation between TNM staging and nutritional impairment indices, and found a significant correlation (r = 0.232, *P* = 0.030).

### Nutrition Counseling

A total of 4,032 participants were confused about nutrition or needing nutrition guidance; only 1,678 had visited the nutrition department for counseling; and only 1,450 patients at nutritional risk had ever consulted the clinical dietitians. When asked about their willingness to obtain nutrition knowledge, most participants (3,897) wanted to be guided by clinicians; only 585 wanted to be guided by dietitians. Furthermore, 2,099 (31.4%), 2,045 (30.59%), 3,089 (46.21%), and 908 (13.58%) patients achieved nutrition knowledge from TV health programs, nutrition books, WeChat official accounts, and nutrition lectures, respectively. Compared with face-to-face offline consultations, these multimedia platforms were more convenient to access.

### The Association Between Influencing Factors and Nutritional Risk

Based on previous analysis, there were significant differences in age, sex, TNM staging, medical insurance, and education between with and without nutritional risk groups. The regression analysis showed that the association between nutritional risk and several factors, including age (OR 1.114, 95%CI 1.106, 1.325), TNM staging III (OR 1.891 95%CI 1.171, 3.916) and IV (OR 2.136, 95%CI 1.054, 4,222), bone marrow transplant (OR 1.427, 95%CI 1.191, 2.901), interval of oncological therapy <1 month (OR 1.472, 95%CI 0.312), and nutritional support (OR 0.497, 95%CI 0.287, 0.812) (Table 3).

**Table 3:** The regression analysis of influencing factors and nutritional risk.

**Variables**	**OR (95% CI)**	***P***
**Age**		
≤ 65 years old	1	0.036
>65 years old	1.114 (1.106, 1.325)	
**Sex**		
Male	1	0.391
Female	0.924 (0.843, 1.412)	
**Payment methods**		
Urban resident medical insurance	1	0.417
Commercial insurance	0.965 (0.834, 1.379)	0.312
Rural cooperative medical insurance	1.413 (0.812, 2.341)	0.712
Self-paid	1.109 (0.918, 1.293)	0.059
**Education**		
Primary school or under	1	0.124
Middle school	0.642 (0.420, 1.012)	0.237
Bachelor's or above	0.142 (0.024, 1.410)	0.062
**TNM staging**		
I	1	0.118
II	1.452 (0.762, 3.462)	0.247
III	1.891 (1.171, 3.916)	0.030
IV	2.136 (1.054, 4.222)	0.037
**Permanent residential**		
Capital city	1	
Prefecture level cities	0.462 (0.302, 1.364)	0.248
Country-level city	0.681 (0.325, 1.572)	0.102
Rural areas	0.642 (0.423, 1.416)	0.174
**Oncological therapy**		
Surgery	1	0.062
Chemotherapy	0.912 (0.791, 1.421)	0.263
Radiotherapy	0.879 (0.364, 1.880)	0.685
Bone marrow transplant	1.427 (1.191, 2.901)	0.037
**Interval of oncological therapy**		
>12 months	1	0.074
6–12 months	1.082 (0.581, 2.012)	0.166
1–6 months	1.266 (0.481, 2.791)	0.104
<1 month	1.472 (1.112, 2.521)	0.030
**Nutritional support**		
No	1	0.014
Yes	0.497 (0.287, 0.812)	

## Discussion

To date, this study is the broadest online survey with the largest sample size to investigate dietary intake and nutritional status of non-hospitalized cancer survivors in China. Being regarded as an early manifestation and an important cause of malnutrition in cancer patients, anorexia was exhibited by 52.7% of patients, thereby presenting with a high prevalence. Anorexia was the main cause for reduced food intake and an important predictor for mortality (18, 19). However, the presence/absence of anorexia was not assessed by nutrition risk screening tools. In fact, patients may present with anorexia but without experiencing significant weight loss due to the administration of artificial nutrition. Therefore, considering anorexia as an early event during cancer progression, its evaluation would be useful in the screening process to early discover of nutritional derangements. The study showed that 31.6% of patients suffered from significant weight loss in the past 3 months and 25.0% had reduced food intake in the past week. In cancer patients weight loss is regarded as an early warning signal of wasting process involving, and an involuntary body weight loss>5% calls for urgently performing a systematic nutritional assessment in cancer (19).

Discover and diagnosis of nutritional risk and malnutrition are crucial in cancer patients (1, 15). NRS-2002 and Malnutrition Universal Screening Tool (MUST) are two of the most popular tools used in clinical practice (20, 21), but there is no consensus on which screening method is more efficient and appropriate in an oncology population (22). NRS-2002 is widely used as a valid nutrition screening tool in clinics for not only general population, but also cancer patients. The Patient-Generated Subjective Global Assessment (PG-SGA), a broadly used for nutrition assessment for cancer patients (6, 23), consists of the patient-generated and the professional component (24). The first part, also regarded as PG-SGA Short Form, is perceived comprehensible and easy (25), and can be completed by the patients or their carers quickly (6), while the professional part, especially the physical examination (25), making it not suitable for online survey. The mini-Nutritional Assessment-Short Form (MNA-SF), in addition to the evaluation of current BMI, weight loss, food intake reduction, burden of disease, investigated the presence of neuropsychological problems and low mobility, however it was specifically designed to evaluate the malnutrition in elders (26). The Global Leadership Initiative on Malnutrition (GLIM) has engaged several global clinical nutrition societies to reach a consensus on the diagnostic criteria of malnutrition in clinical settings (27). A study conducted in cancer patients to compare different nutrition risk screening tools found NRS-2002 was better correlated with the GLIM criteria than MUST and PG-SGA, and could serve as a good candidate for first-step malnutrition risk screening according to the GLIM diagnostic scheme. Although the PG-SGA is a sensitive tool to detect compromised nutritional status, the assessment had a low specificity in the diagnosis of malnutrition according to the GLIM criteria (28).

In this study, the overall prevalence of nutritional risk was 33.9%, as screened by NRS-2002; this was lower than the 40.2 and 50% reported among the Chinese population (28, 29). This may be explained by the fact that the participants mentioned above were hospitalized cancer patients, whose disease might be of high severity or who were currently under treatment. However, in this study, the participants were either in remission or between treatment cycles, thereby rendering less treatment effects or disease severity. Even so, the nutritional risk of this population was still high and should not be ignored; even though tumor burden is not severe, appropriate and early nutritional intervention can help achieve a clinical benefit.

Nutritional risk varied according to personal characteristics. The NRS-2002 scores tended to increase with age, which is similar to previous study (30). Elderly patients with cancer are more prone to nutritional problems due to organ dysfunction and reduced treatment tolerance. Nevertheless, for some types of cancer in our study, such as gastric, esophageal, skin, and breast cancers, young and middle-aged patients showed higher NRS-2002 scores. This may be related to the higher malignancy of tumors that occur in young and middle-aged patients. Meanwhile, the treatment plan for this population may also be more aggressive, thus leading to a greater impact on gastrointestinal symptoms and food intake. Besides, our study showed the nutritional risk of this population is gradually deteriorating with malignancy of tumor, especially more than stage III. Moreover, we found that the NRS-2002 scores significantly differed according to education level and medical insurance type. Cancer patients with higher education levels or commercial insurance probably intend to perform early detection and interventions, suggesting an imbalance of medical resources at present.

Interestingly, for most non-digestive cancer survivors, a higher NRS-2002 score seemed to be associated with a higher TNM stage, which was comparable to the result of the largest investigation of hospitalized cancer patient (11). However in subgroup analysis of our study, no significant difference was found between TNM stages in digestive system cancers. This indicates that digestive system cancers could influence food intake and body weight even at an early stage, thus leading to increased nutritional risk. Therefore, nutritional risk screening should be performed as early as possible. The NRS-2002 score was higher in patients who had combined surgery plus radio/chemotherapy, followed by those received surgery or radio/chemotherapy alone, thus suggesting that extra attention should be paid to this population.

In the regression analysis, age, TNM staging, oncological therapy, and time interval of treatment were associated with NRS-2002, and also nutritional impairment score was close correlated with TNM staging, indicating the intimate relationship between nutritional risk and disease. However, the association between nutritional risk and the demographic characteristics was not found, it might be the complicated relationship among these factors.

Patients spend the most time at home or in community sanatoriums, especially in the era of COVID-19, patients had fewer chances to visit a hospital for nutrition counseling. However, the nutritional problems of these non-hospitalized patients were distressingly undertreated. A large number of the participants had nutrition-related queries, but only a minority received professional guidance or intervention from dietitians. A French study found that only 35.8% of cancer patients received regular nutrition counseling; of which, 56.3% were provided by nutritionists or dietitians, 31.9% by doctors, and 11.8% by other medical staff (31). A Chinese study included 1,138 cancer patients and found merely 14% of them had cancer counseling (32). This shows that there are still gaps in the standardized treatment as well as in clinical practice. In addition, the importance of dietitians or nutritional support is not yet fully recognized by clinicians or patients (33–35). This may be due to the insufficient participation of dietitians during oncologic treatments or lack of collaboration between oncologists and clinical nutritionists (36), where nutritional intervention is not routinely included in clinical practice (37).

Nutritional support benefits patients with nutritional risk as to improving clinical outcomes (38); conversely, it may not help but increase the costs for those with no risk. A Chinese study focusing on hospitalized gastric cancer patients found that 59.1% of patients with malnutrition did not receive nutritional support, while 25.5% at no risk were given needless intervention (39). Only 30–60% of cancer patients with nutritional risk were provided with nutritional support (32, 40). In this study, a higher proportion of patients with and without nutritional risk received support, indicating the inappropriate use of nutritional intervention. Besides, nutrition support, such as ONS, nasal enteral nutrition, and parenteral nutrition were used by some non-hospitalized patients, but the appropriateness of the application was not fully evaluated, and whether the nutrition requirement was met remained unclear.

Few studies have focused on the composition of nutritional risk screening to identify the main contributors to NRS-2002. The proportion of nutritional impairment score ranged from 50–70% in respiratory system, bone, and nervous system cancers; however, they were mainly contributed by weight loss, not dietary reduction. The incidence of weight loss and dietary reduction was high in digestive system, nervous system, and bone cancers, thus indicating that the nutritional status of these patients was seriously impaired. For those with nutritional impairment, proper nutritional intervention can improve the nutritional status. However, for those with more severe diseases, aggressive therapies for primary cancer may bring more benefits. Therefore, in addition to paying attention to the existence of nutritional risk, the composition of NRS-2002 is also important for dietitians in choosing the appropriate intervention. We further found the close correlation between TNM and nutrition impairment, indicating that disease and nutrition impairment have influence on each other and should be paid attention.

Given the patients' intention to acquire nutritional knowledge and modern communication technology, results of this study suggest that online survey is a convenient and quick method to delivery nutritional risk screening for cancer survivors. Several web-based lifestyle or psychological interventions for cancer patients were conducted and shown acceptable and feasible by patients (41, 42). More important, online survey can reach non-hospitalized patients, so that nutrition support can be integrated into patients' daily life more deeply and permanently. Compared to the largest survey of Chinese hospitalized cancer patients conducted between 2013 to 2020 and enrolled 47,448 patients from 22 provinces (10), our study enrolled 6,648 valid surveys covering 31 provinces, autonomous regions, and municipalities within 6 months, suggesting it is efficient. Strict quality control was performed during the entire process. The “impaired nutritional status” part of the NRS-2002 included age, recent food intake, and weight change; these can be easily reported by the patients through an online questionnaire. The “severity of disease” might be difficult to assess; however, it can be evaluated by the site and staging of the tumor as well as the recent therapy methods reported by the subjects. Therefore, NRS-2002 can be conducted online; this study provides evidence for an online-based NRS-2002 evaluation.

This study had several limitations. First, the NRS-2002 score is qualitative and simple; therefore, it may not be possible to comprehensively evaluate complex nutritional problems. Second, self-report measures may result in biased estimates; however, these are presumably equally distributed among all participants. Thirdly, the online survey may lose sight of people who are incapable to use cellphone, which is a common problem of this method. Lastly, since the limitation of online survey, whether the nutrition requirement was meet by nutritional support cannot be known because physical examination and medical status evaluation cannot be done, which is important in nutrition care practice. Therefore, further studies are warranted to assess the nutritional status of non-hospitalized survivors based on more objective and abundant data measures and to determine their long-term clinical outcomes.

## Conclusions

This study found a large proportion of these population presenting dietary intake reduction, weight loss, and high nutritional risk, and regular monitoring and assessment follow-up system should be established to evaluate the nutritional status of non-hospitalized cancer survivors. Moreover, based on our practice, online survey may be a convenient and suitable method for nutrition status investigation. Medical staff must be aware of the nutritional risks, the contributors of the NRS-2002 score, and provide nutrition intervention for non-hospitalized cancer survivors to improve nutritional status and clinical outcomes. Lastly, due to the low percentage of nutrition consultation achieved, we recommend professional nutrition consultation and education should be carried out in clinical practice.

## Data Availability Statement

The raw data supporting the conclusions of this article will be made available by the authors, without undue reservation.

## Ethics Statement

The studies involving human participants were reviewed and approved by Human Ethics Committee of Peking Union Medical College Hospital. The patients/participants provided their written informed consent to participate in this study.

## Author Contributions

FW and KY were responsible for the design of the study, revised the paper, and provided feedback. FW, KY, QD, R-rL, and J-yG conducted the research and analyzed the data. FW, QD, and C-wL wrote the manuscript. All authors contributed to the article and approved the submitted version.

## Conflict of Interest

The authors declare that the research was conducted in the absence of any commercial or financial relationships that could be construed as a potential conflict of interest.

## Publisher's Note

All claims expressed in this article are solely those of the authors and do not necessarily represent those of their affiliated organizations, or those of the publisher, the editors and the reviewers. Any product that may be evaluated in this article, or claim that may be made by its manufacturer, is not guaranteed or endorsed by the publisher.
