# Supplementary material for: Nutrition Risk Screening and Related Factors Analysis of Non-hospitalized Cancer Survivors: A Nationwide Online Survey in China
Source: Front Nutr. 2022 Jun 21;9:920714. doi: 10.3389/fnut.2022.920714 (PMC9253613; doi:10.3389/fnut.2022.920714)
Supplement: Supplementary file 1 [file Table_1.DOCX]

**Supplementary Table.** Demographic characteristics of the subjects

| Variables | Proportion (%) | Variables | Proportion (%) |
| --- | --- | --- | --- |
| Age | | Gender | |
| <45y | 19.6 | Male | 37.8 |
| 45-64.9y | 66.7 | Female | 62.2 |
| 65-85y | 13.6 | Insurance | |
| >85y | 0.1 | Urban medical insurance for employee | 61.2 |
| NRS-2002 score | | Urban medical insurance for resident | 10.6 |
| <3 | 66.1 | Rural cooperative medical insurance | 25.9 |
| ≥3 | 33.9 | Self-pay | 1.1 |
| TNM staging | | Unsure | 1.2 |
| I | 16.3 | Education level | |
| II | 22.8 | Primary school or under | 11.8 |
| III | 22.2 | Middle school | 61.2 |
| IV | 27.7 | Bachelor’s degree or above | 27.0 |
| Unsure | 8.9 | Anti-tumor treatment method | |
| Other staging | 2 | Surgery | 15.9 |
| Location | | Radio/chemotherapy | 30.9 |
| Provincial capital | 33.5 | Surgery+ Radio/chemotherapy | 35.3 |
| Prefecture-level city | 29.8 | Others | 17.9 |
| County-level cities | 5.9 |  |  |
| Rural | 30.8 |  |  |
| BMI | |  |  |
| <18.5kg/m2 | 8.8 |  |  |
| 18.5-23.9 kg/m2 | 56.4 |  |  |
| 24-27.9 kg/m2 | 25.0 |  |  |
| ≥28 kg/m2 | 8.3 |  |  |


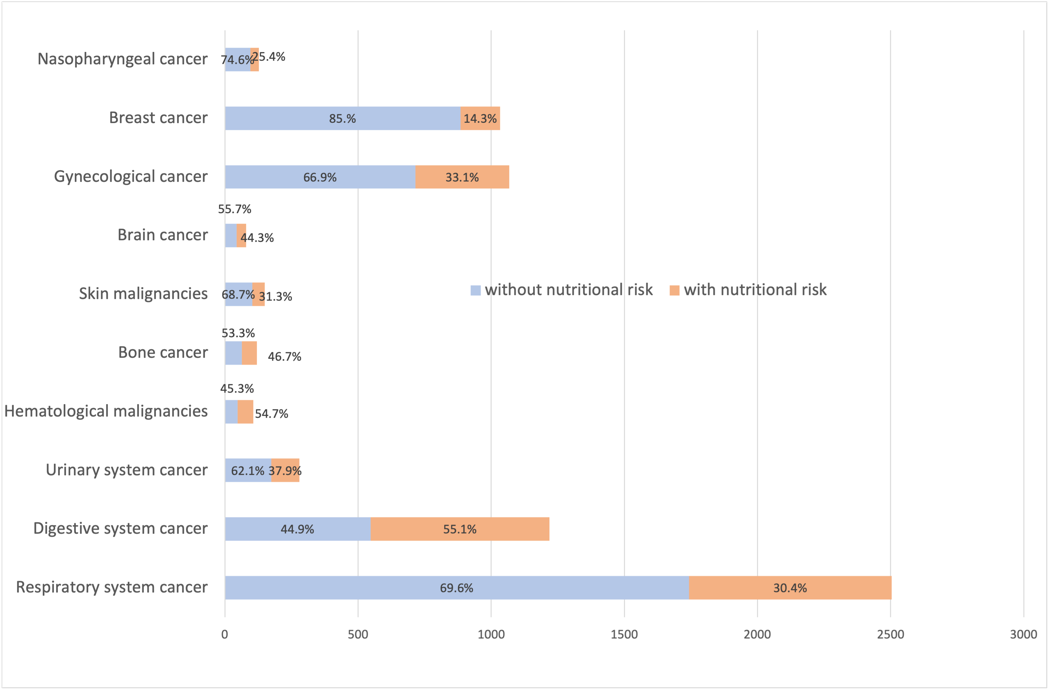


**Figure 1.** The proportion of patients with nutrition risk of different cancer type
